# Opioid-Mediated Astrocyte–Neuron Signaling in the Nucleus Accumbens

**DOI:** 10.3390/cells8060586

**Published:** 2019-06-14

**Authors:** Michelle Corkrum, Patrick E. Rothwell, Mark J. Thomas, Paulo Kofuji, Alfonso Araque

**Affiliations:** Department of Neuroscience, University of Minnesota, Minneapolis, MN 55455, USA; mcorkrum@umn.edu (M.C.); rothwell@umn.edu (P.E.R.); tmhomas@umn.edu (M.J.T.)

**Keywords:** astrocytes, opioids, synaptic transmission

## Abstract

Major hallmarks of astrocyte physiology are the elevation of intracellular calcium in response to neurotransmitters and the release of neuroactive substances (gliotransmitters) that modulate neuronal activity. While μ-opioid receptor expression has been identified in astrocytes of the nucleus accumbens, the functional consequences on astrocyte–neuron communication remains largely unknown. The present study has investigated the astrocyte responsiveness to μ-opioid signaling and the regulation of gliotransmission in the nucleus accumbens. Through the combination of calcium imaging and whole-cell patch clamp electrophysiology in brain slices, we have found that μ-opioid receptor activation in astrocytes elevates astrocyte cytoplasmic calcium and stimulates the release of the gliotransmitter glutamate, which evokes slow inward currents through the activation of neuronal N-methyl-D-aspartate (NMDA) receptors. These results indicate the existence of molecular mechanisms underlying opioid-mediated astrocyte–neuron signaling in the nucleus accumbens.

## 1. Introduction

Opioids are efficacious compounds used as analgesics in the medical setting [1,2]; however, opioids are also deadly addictive substances, due to their rewarding actions in the brain [3]. Opioid substance use disorder has grown exponentially in the past decades, resulting in tens of thousands of overdose deaths [4]. Opioids are both naturally occurring and synthetically derived substances, such as morphine, heroin, oxycodone and fentanyl. The rewarding and addicting effects of opioids are mediated by their activation of inhibitory G-protein coupled receptors (G_i/o_) [5]. There are three main subtypes of opioid receptors: μ, δ, and κ receptors [5].

While the majority of research on opioids has focused on neurons, very little is known about the involvement of astrocytes in opioid signaling. Recent evidence demonstrates the expression of μ-opioid receptors on astrocytes in the hippocampus, ventral tegmental area, and the nucleus accumbens [6]. The μ-opioid receptors localized to astrocytes in the hippocampus were found to evoke astrocytic glutamate release through TREK-1-containing, two-pore potassium (K2P) channels [7]. In addition, opioid receptor activation in cultured astrocytes has been shown to increase intracellular calcium via inositol-1,4,5-trisphosphate (IP_3_) signaling [8]. However, whether astrocytes in situ respond to opioid signaling in the nucleus accumbens, the primary brain area involved in reward and addiction remains unknown. 

Traditionally, astrocytes have been considered to be support cells of the brain, contributing to ion homeostasis, maintaining the blood–brain barrier, and providing trophic and metabolic support to neurons [9]. However, accumulating evidence indicates that astrocytes contribute to brain information processing through functional signaling interaction with neuronal synaptic elements, establishing tripartite synapses [9]. A single astrocyte can make synaptic contacts with tens of thousands of synapses, and over 50% of excitatory synapses in the hippocampus exhibit close proximity to an astrocyte process [10]. Astrocytes express a large variety of neurotransmitter receptors, which bind neurotransmitters and eventually result in an increase in cytoplasmic calcium and the release of neuroactive substances (termed gliotransmitters) that modulate neuronal activity, synaptic transmission, and plasticity [11,12,13,14]. 

Using brain slices, we have investigated whether nucleus accumbens astrocytes respond to opioids and whether opioid signaling in astrocytes stimulates gliotransmission. Since opioids are known to exert both their analgesic and rewarding effects via activation of μ-opioid receptors, we focused the present study on this subtype of opioid receptors. We have found that astrocytes respond to μ-opioid receptors with intracellular calcium elevations. These calcium elevations are associated with the release of glutamate that evokes NMDAR-mediated slow inward currents (SICs) in neurons. These results indicate that astrocytes participate in opioid signaling in the nucleus accumbens.

## 2. Materials and Methods

### 2.1. Ethics Statement 

All animal care and sacrifice procedures were approved by the University of Minnesota Institutional Animal Care and Use Committee (IACUC), with compliance to the National Institutes of Health guidelines for the care and use of laboratory animals.

### 2.2. Animals

Mice were housed under 12/12-h light/dark cycle and up to five animals per cage. The following animals (males and females) were used for the present study: C57BL/6J, IP_3_R2^−/−^ (donated by Dr. J. Chen), and Oprm1 flox/flox (MOR flox/flox; Jackson stock # 030074). All animals were at least 4 weeks of age at time of experiment. 

### 2.3. Stereotaxic Surgery

Animals at least 4 weeks of age were anesthetized with a ketamine (100 mg/kg)/xylazine (10 mg/kg) cocktail. Viral vectors (1 μL) were injected bilaterally with a Hamilton syringe attached to a 29-gauge needle at a rate of 0.8–1.25 microliters/minute. The following coordinates were used to target the nucleus accumbens (NAc) core: (anterior–posterior (AP): +1.50 mm; medial–lateral (ML): +/−0.75 mm; dorsal–ventral (DV): −4.50 mm). To visualize astrocyte calcium levels, the AAV5-GfapABC1D-cytoGCaMP6f-SV40 (Penn Vector Core) viral vector was targeted to astrocytes and not neurons (only 1.1% of NeuN-expressing neurons colocalized with GCaMP6-expressing cells). To target μ-opioid receptors in the astrocytes, the AAV8–GFAP–mCherry–CRE viral vector (UNC vector core) was targeted to the NAc core of MOR flox/ flox mice. For astrocyte visualization, AAV8-GFAP-mCherry (UMN vector core) was targeted to nucleus accumbens core astrocytes. 

### 2.4. Nucleus Accumbens Core Slice Preparation

Animals were anesthetized with isoflurane and intracardially perfused with ice-cold artificial cerebral spinal fluid (ACSF) containing (in mM) NaCl 124, KCl 2.69, KH_2_PO_4_ 1.25, MgSO_4_ 2, NaHCO_3_ 26, CaCl_2_ 2, and glucose 10, and was oxygenated with 95% O_2_/5% CO_2_ (pH = 7.3–7.4). Mice were then decapitated, and the brain was extracted. Brain slices containing the nucleus accumbens core (350 μm) were obtained using a vibratome (Leica VT1200S) and recovered at room temperature for at least 30 min in oxygenated ACSF. Slices were transferred to an immersion recording chamber and superfused (2 mL/min) with oxygenated ACSF, and cells were visualized with an Olympus BX50WI microscope (Olympus Optical, Tokyo, Japan) or Leica SP5 multi-photon microscope. 

### 2.5. Electrophysiology

Whole-cell-patch clamp electrophysiology recordings were made from NAc core neurons. For slow inward current recordings, the ACSF composition was the following (in mM): NaCl 124, KCl 2.69, KH_2_PO_4_ 1.25, NaHCO_3_ 26, CaCl_2_ 4, glucose 10, and glycine 10 μM. No magnesium was included in the solution, in order to optimize activation of NMDA receptors, and tetrodotoxin (TTX) (1 μM) was included to block sodium-dependent action potentials. The neuronal internal solution consisted of (in mM) KMeSO_4_ 135, KCl 10, HEPES 10, NaCl 5, ATP–Mg^+2^ 2.5, and GTP–Na^+^ 0.3 (pH = 7.3); when filled, patch electrodes had a resistance of 3–10 MΩ. Neurons were held at a membrane potential of −70 mV. Recordings were made with PC-ONE amplifiers (Dagan Instruments, Minneapolis, MN, USA) and fed to a Pentium-based PC through a DigiData 1440A interface board. Signals were filtered at 1 KHz and acquired at a 10 KHz sampling rate. Data display, acquisition, and storage was conducted utilizing the pCLAMP 10.4 (Molecular Devices, Sunnyvale, CA, USA) software. 

### 2.6. Ca^2+^ Imaging

Astrocyte cytoplasmic calcium levels in the NAc core were examined utilizing epifluorescence (CCD camera; Hammamatsu, Japan) and multi-photon microscopy (Leica SP5 multi-photon microscope; Leica Microsystems, IL, USA). Cells were illuminated with a 490 nm LED and images were obtained at a 1 Hz frequency. For epifluorescence imaging, The LED and the CCD camera were controlled and synchronized by the MetaMorph software (Molecular Devices). For multi-photon imaging, the Leica SP5 multi-photon microscope was controlled by the Leica LAS software.

### 2.7. Drugs and Drug Application

DAMGO ([D-Ala^2^, NMe-Phe^4^, Gly-ol^5^]-enkephalin) was purchased from Tocris Bioscience (MN, USA). For exogenous DAMGO (500 μM) application, the drug was dissolved in double distilled water; a borosilicate glass pipette containing the drug was placed over the NAc core, and the drug was applied with a pressure pulse (0.5 bar, 60 s). Octahydro-12-(hydroxymethyl)-2-imino-5,9:7,10a-dimethano-10a*H*-[1,3]dioxocino [6,5-*d*] pyrimidine-4,7,10,11,12-pentol (tetrodotoxin (TTX)) was purchased from Tocris Bioscience. All other drugs were purchased from Sigma (St. Louis, MO, USA). 

### 2.8. Data Analysis

Data were analyzed using MATLAB (MathWorks, Natick, MA, USA) and SigmaPlot 12.5 (Systat Software Inc, San Jose, CA, USA). Custom MATLAB code (MATLAB R2018; MathWorks) was developed to semi-automatically analyze calcium imaging and slow inward current data. Slow inward current detections were confirmed manually for each experiment. Ca^2+^ variations of astrocytes were estimated as changes of the fluorescence signal over the baseline (ΔF/F_0_), and astrocytes were considered to show a Ca^2+^ event when the ΔF/F_0_ increase was at least two times the standard deviation of the baseline. Relative Ca^2+^ events were determined by normalizing events that occur 30 s before DAMGO application and 30 seconds after the initiation of DAMGO application. 

### 2.9. Immunohistochemistry

Animals were anesthetized with Avertin (2,2,2 tribromoethanol, 240 mg/kg, i.p.) and intracardially perfused with ice cold phosphate buffered saline (PBS), and subsequently with 4% paraformaldehyde (PFA) in 0.1 M phosphate buffered saline (pH 7.4). The brain was removed and 100 μm coronal sections were made using a Leica VT1000S vibratome. Vibratome sections were incubated overnight in blocking buffer (0.1% Triton X-100, 10% donkey or goat serum in PBS) at room temperature. The primary antibodies were diluted in the blocking solution, and the sections were incubated for 48 h at 4 °C. The following primary antibodies were used: mouse anti-NeuN (MilliporeSigma, Burlington, MA, USA, 1:500), rabbit anti-GFAP (Sigma, 1:500), chicken anti-mCherry (Abcam, Cambridge, MA, USA, 1:500), and rabbit anti-MOR (Neuromics, Edina, MN, USA, 1:200). The slices were washed three times for thirty minutes each in 0.1 M PBS. The secondary antibodies were diluted in the secondary antibody buffer (0.1% Triton X-100, 5% Donkey or Goat serum in PBS) and incubated overnight at 4 °C. The following secondary antibodies were used: AlexaFluor 405 goat anti-mouse (Invitrogen, 1:500), AlexaFluor 488 goat anti-rabbit (Invitrogen, NH, USA, 1:1000), and AlexaFluor 594 goat anti-chicken (Invitrogen, 1:1000). The sections were then washed three times with 1x PBS for thirty minutes each, and mounted using Vectashield Mounting media (Vector Laboratories, Burlingame, CA, USA). Mounted slices were imaged using an Olympus Fluoview 1000 microscope.

### 2.10. Statistics

Data are expressed as mean ± standard error of the mean (SEM). Results were compared using a two-tailed Student’s paired *t*-test or Wilcox Signed Rank Test (α = 0.05). Statistical differences were established with *p* < 0.05 (*), *p* < 0.01 (**), and *p* < 0.001 (***).

## 3. Results

**Figure 1:**
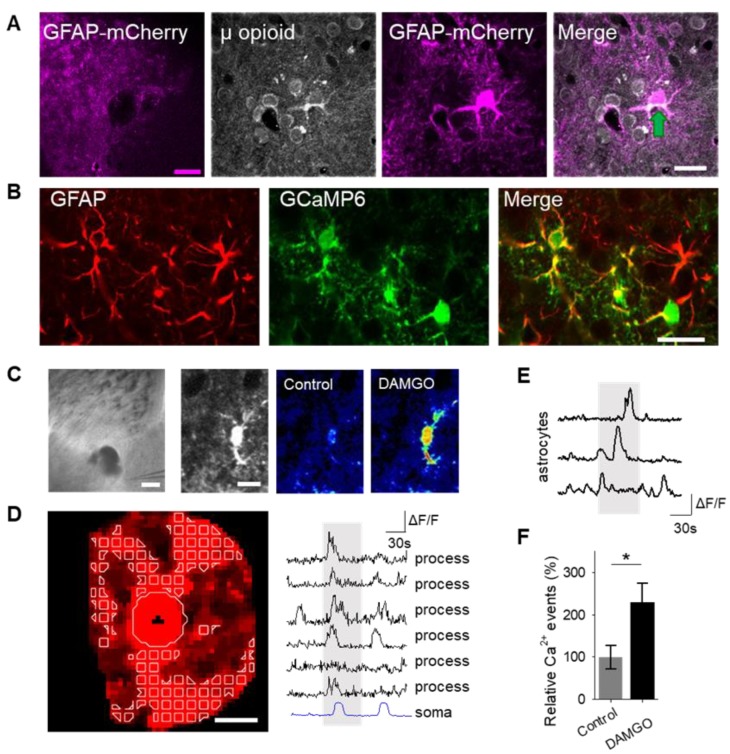
μ-Opioid receptor activation increases astrocyte cytoplasmic calcium levels. (**A**) Immunohistochemical images of μ-opioid receptor expression in mCherry-expressing astrocytes and the merged image in the nucleus accumbens. Scale bar: magenta = 200 μm; white = 20 μm. Green arrow indicates colocalization. (**B**) Immunohistochemical images of GCaMP6 expression in Glial fibrillary acidic protein (GFAP)-expressing cells in the nucleus accumbens. Scale bar = 20 μm. (**C**) Image (scale bar = 200 μm) illustrating experimental approach of locally targeting DAMGO ([D-Ala^2^, NMe-Phe^4^, Gly-ol^5^]-enkephalin) to the nucleus accumbens, and pseudocolor images (scale bar = 10 μm) showing the fluorescence intensities of a GCaMP6-expressing astrocytes before and after DAMGO application in the nucleus accumbens. (**D**) Representative image of the soma and process regions of interests (sale bar = 10 μm), as well as calcium traces of nucleus accumbens astrocyte processes and the soma (gray shading indicates DAMGO application). (**E**) Calcium traces of astrocytes (gray shading indicates DAMGO application). (**F**) Relative calcium events before (control) and after DAMGO application. Data are expressed as mean ± standard error of the mean (SEM), * *p* < 0.05.

To examine the role of astrocytes in μ-opioid signaling in the nucleus accumbens, we aimed to confirm that astrocytes in this region expressed μ-opioid receptors [6]. Immunohistochemistry experiments demonstrated μ-opioid receptor expression in astrocytes in the nucleus accumbens core (Figure 1A; Table 1). 

Astrocyte excitability manifests as cytoplasmic calcium elevations [15,16,17]; therefore, we aimed to test the hypothesis that the selective μ-opioid receptor agonist, DAMGO, would increase astrocyte calcium levels. To monitor astrocyte calcium, we used the genetically encoded calcium indicator (GCaMP6f) selectively expressed in NAc core astrocytes (Figure 1B). Local application of DAMGO with a micropipette (500 μM, 60 s, 0.5 bar) increased cytoplasmic calcium levels in both the soma and in the processes of astrocytes (Figure 1C,D). We found that astrocyte calcium events significantly increased after DAMGO application (*n* = 257 astrocytes from *n* = 19 experimental planes of view, *p* = 0.02; Figure 1E,F; Table 3) when compared to baseline levels, indicating that astrocytes respond to μ-opioid signaling with increases in calcium. 

**Figure 2:**
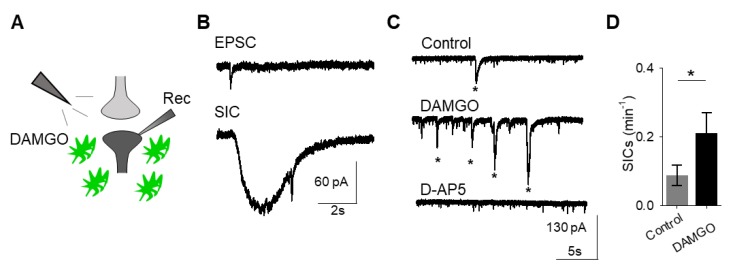
μ-Opioid receptor activation evokes slow inward currents in neurons. (**A**) Scheme of electrophysiology experimental approach illustrating local application of DAMGO to the nucleus accumbens and recording of nucleus accumbens medium spiny neurons. (**B**) Representative traces of excitatory post-synaptic potential (EPSC; top) and slow inward current (SIC; bottom). (**C**) Representative electrophysiology recordings in control conditions (top), after DAMGO application (middle), and in the presence of the D-AP5 (bottom). Stars indicate SICs. (**D**) SICs per minute before (control) and after DAMGO application. Data are expressed as mean ± SEM, * *p* < 0.05.

A consequence of astrocyte calcium elevations is the release of neuroactive substances, such as glutamate, which modulate neuronal activity [18]. Astrocyte glutamate has been shown to activate extrasynaptic NMDA receptors to produce slow inward currents (SICs) in multiple brain areas [19,20,21]. SICs are distinct from excitatory post-synaptic currents, due to their slower time course and sensitivity to the NMDA receptor antagonist D-AP5 (50 μM; Figure 2A–C). We recorded whole-cell currents in neurons in the presence of tetrodotoxin (TTX), in order to block sodium-dependent action potentials, before, during, and after applying DAMGO. We found that in addition to modulating astrocyte calcium, local delivery of DAMGO increased the frequency of SICs (*n* = 17, *p* = 0.01; Figure 2C,D; Table 3), indicating that μ-opioid signaling in astrocytes stimulates the release of astrocytic glutamate.

**Figure 3:**
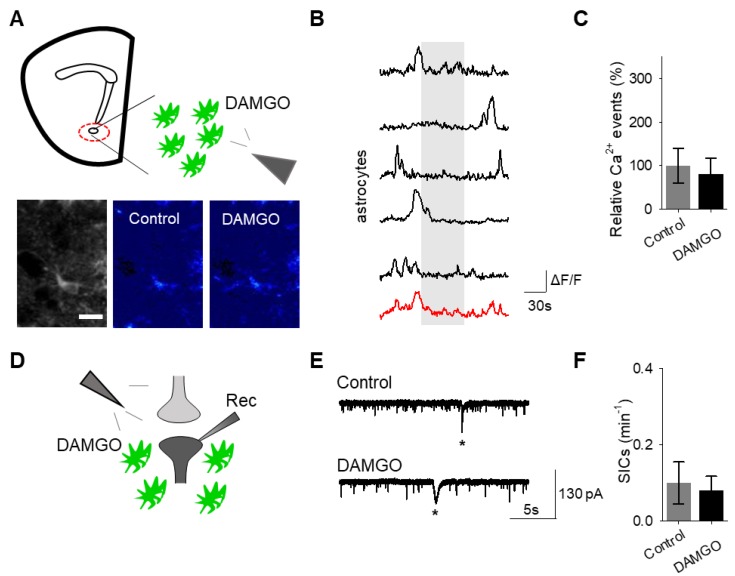
The opioid receptor antagonist naltrexone attenuates DAMGO actions on astrocytes and neurons. (**A**) Scheme of experimental approach and pseudocolor images showing the fluorescence intensities of GCaMP6-expressing astrocytes in the nucleus accumbens, before and after DAMGO application in the presence of naltrexone (10 μM). (**B**) Calcium traces of astrocytes (gray shading indicates DAMGO application; red trace = average) in the presence of naltrexone. (**C**) Relative calcium events before (control) and after DAMGO application in the presence of naltrexone. (**D**) Scheme of electrophysiology experimental approach in the nucleus accumbens. (**E**) Representative electrophysiology recordings in control conditions and after DAMGO application in the presence of naltrexone. (**F**) SICs per minute before (control) and after DAMGO application in the presence of naltrexone. Data are expressed as mean ± SEM, scale bar = 10 μm.

To confirm that the effects of DAMGO were mediated by the activation of opioid receptors, we conducted experiments in the presence of the global opioid receptor antagonist naltrexone (10 μM). We found that in the presence of naltrexone, DAMGO no longer induced astrocytic calcium elevations (*n* = 55 astrocytes from *n* = 7 experimental planes of view, *p* = 0.77; Figure 3A–C; Table 3) nor affected neuronal SICs (*n* = 5, *p* = 0.62; Figure 3D–F; Table 3), suggesting that DAMGO actions are via opioid receptors to modulate astrocyte calcium signaling and the generation of neuronal SICs.

**Figure 4:**
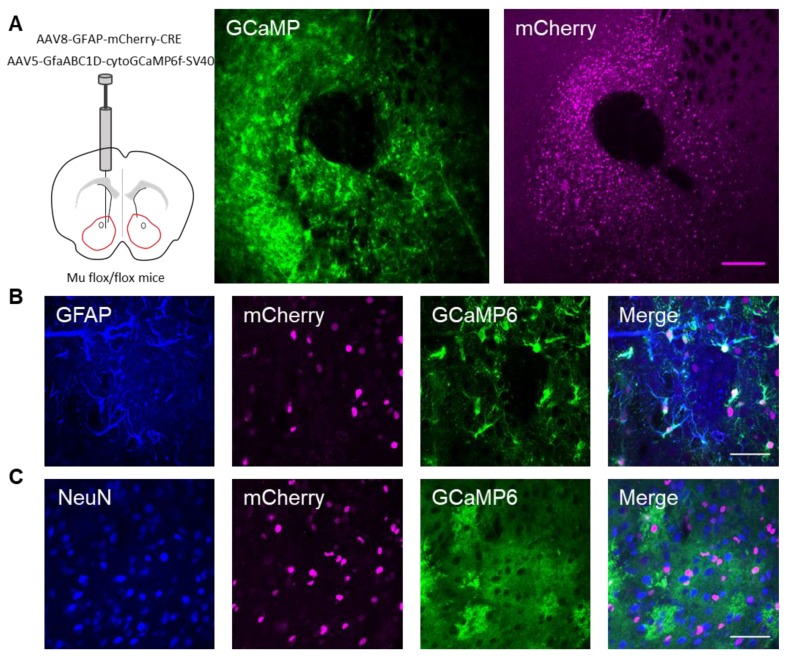
Targeting AAV8–GFAP–mCherry–CRE to astrocytes in the nucleus accumbens of μ-flox mice. (**A**) Scheme of viral targeting experimental approach and immunohistochemistry images of GCaMP6 (green) and AAV8–GFAP–mCherry–CRE (magenta) in the nucleus accumbens. (**B**) Immunohistochemistry images of mCherry^+^ cells co-stained with GFAP and GCaMP6, and the merge image in the nucleus accumbens. (**C**) Immunohistochemistry images of mCherry^+^ cells co-stained with NeuN and GCaMP6, and the merge image in the nucleus accumbens. Magenta scale bar = 200 μm, white scale bar = 50 μm.

**Figure 5:**
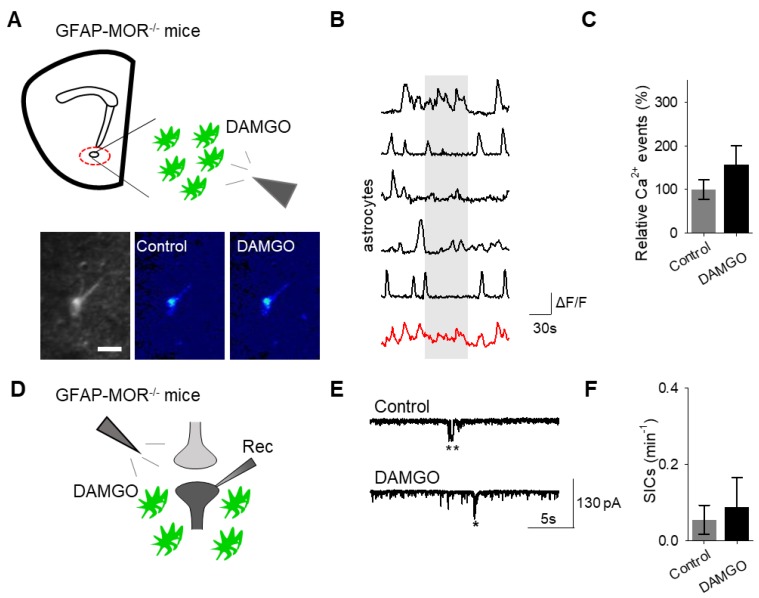
Astrocyte μ-opioid receptors mediate DAMGO actions on astrocytes and DAMGO-evoked neuronal slow inward currents. (**A**) Scheme of experimental approach and pseudocolor images showing the fluorescence intensities of GCaMP6-expressing astrocytes in the nucleus accumbens, before and after DAMGO application in slices from GFAP-MOR^−/−^ mice. (**B**) Calcium traces of astrocytes (gray shading indicates DAMGO application; red trace = average) in slices from GFAP- MOR^−/−^ mice. (**C**) Relative calcium events before (control) and after DAMGO application in slices from GFAP- MOR^−/−^ mice. (**D**) Scheme of electrophysiology experimental approach in slices containing the nucleus accumbens from GFAP-MOR^−/−^ mice. (**E**) Representative electrophysiology recordings in control conditions and after DAMGO application in slices from GFAP-MOR^−/−^ mice. (**F**) SICs per minute before (control) and after DAMGO application in slices from GFAP-MOR^−/−^ mice. Data are expressed as mean ± SEM, scale bar = 10 μm.

Next, we investigated whether the actions of DAMGO were mediated by μ-opioid receptors expressed on astrocytes. We generated NAc core GFAP–MOR^-/-^ mice via targeting the viral vector, AAV8–GFAP–mCherry–CRE, to transgenic mice with a floxed μ-opioid receptor gene (MOR flox/flox mice; Figure 4A). We confirmed that the AAV8–GFAP–mCherry–CRE viral vector was targeted to GFAP- and GCaMP6f-expressing astrocytes (Figure 4B; Table 2). Additionally, we confirmed that the AAV8–GFAP–mCherry–CRE was not predominantly targeted to NeuN-expressing neurons (Figure 4C; Table 2). Next, we conducted calcium imaging experiments, and observed no significant astrocyte calcium elevations in response to DAMGO (*n* = 92 astrocytes form *n* = 8 experimental planes of view, *p* = 0.21; Figure 5A–C; Table 3), suggesting that DAMGO acted directly on μ-opioid receptors expressed on astrocytes to produce calcium increases. Additionally, in the NAc core of GFAP-MOR^−/−^ mice we did not observe DAMGO-induced SIC elevations (*n* = 9, *p* = 0.78; Figure 5D–F; Table 3), indicating that the DAMGO-evoked gliotransmission was mediated by μ-opioid receptors expressed on astrocytes.

**Figure 6:**
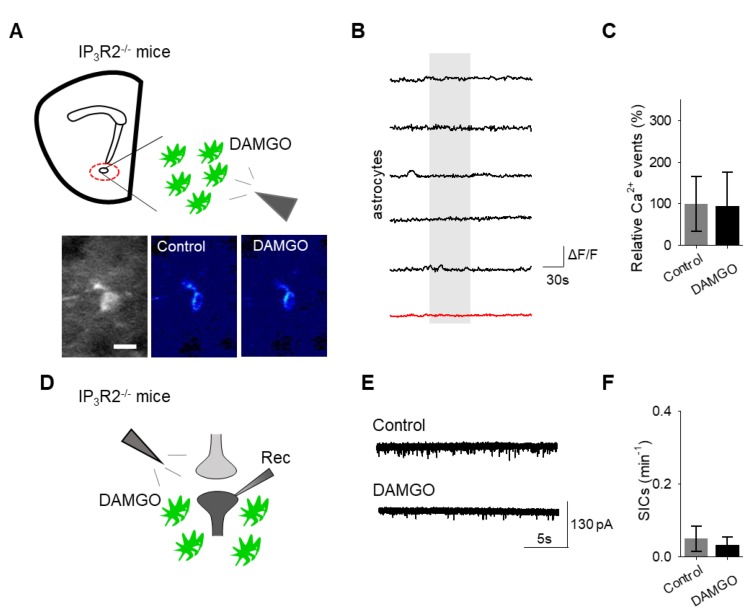
IP_3_ signaling in astrocytes mediates DAMGO actions on astrocytes and neuronal slow inward currents. (**A**) Scheme of experimental approach and pseudocolor images showing the fluorescence intensities of a GCaMP6-expressing astrocytes in the nucleus accumbens before and after DAMGO application in slices from IP_3_R2^−/−^ mice. (**B**) Calcium traces of astrocytes (gray shading indicates DAMGO application; red trace = average) in slices from IP_3_R2^−/−^ mice. (**C**) Relative calcium events before (control) and after DAMGO application in slices from IP_3_R2^−/−^ mice. (**D**) Scheme of the electrophysiology experimental approach in slices containing the nucleus accumbens from IP_3_R2^−/−^ mice. (**E**) Representative electrophysiology recordings in control conditions and after DAMGO application in slices from IP_3_R2^−/−^ mice. (**F**) SICs per minute before (control) and after DAMGO application in slices from IP_3_R2^−/−^ mice. Data are expressed as mean ± SEM, scale bar = 10 μm.

Finally, we investigated the intracellular signaling cascade that mediates the astrocyte responsiveness to DAMGO and the subsequent gliotransmission. A primary pathway of astrocyte calcium signaling is via IP_3_-mediated calcium elevations, governed by the activation of IP_3_ receptors on the endoplasmic reticulum to induce the release of internal calcium stores [15]. We examined the contribution of IP_3_ signaling using IP_3_R2^−/−^ mice, which have impaired G protein-coupled receptor-mediated astrocyte calcium signaling [22,23,24]. No significant changes in astrocyte calcium signaling (*n* = 54 astrocytes form *n* = 8 experimental planes of view, *p* = 0.78 Figure 6A–C; Table 3) and SIC frequency (*n* = 6, *p* = 0.74 Figure 6D–F; Table 3) were evoked by DAMGO in IP_3_R2^−/−^ mice, indicating that DAMGO-evoked astrocyte calcium increases and neuronal SICs were mediated by IP_3_ signaling in astrocytes. 

## 4. Discussion

The current study demonstrates that astrocytes in the nucleus accumbens core express μ-opioid receptors and respond to μ-opioid receptor activation with increases in cytoplasmic calcium. Furthermore, the μ-opioid signaling in astrocytes stimulates the release of the gliotransmitter glutamate, which activates neuronal NMDARs, as evidenced by the increase in the frequency of neuronal SICs. Indeed, the astrocytic increases in calcium and neuronal SICs were evoked by opioid receptors, as evidenced by the attenuation of responses in the presence of the global opioid receptor antagonist naltrexone. Specifically, DAMGO-induced astrocyte calcium increases, and SICs were dependent on calcium release of internal stores via activation of IP_3_R2s, given that the evoked calcium responses and evoked SICs were no longer present in IP_3_R2^−/−^ mice. Additionally, the same effects were seen upon selective genetic ablation of μ-opioid receptors to nucleus accumbens astrocytes. Taken together, these data obtained with pharmacological and transgenic approaches, suggest that astrocytes are key players in opioid signaling in the nucleus accumbens.

The present study further advances the literature on the consequences of opioid signaling on astrocytic function. Past research showed that in response to opioids, such as morphine, astrocytes exhibit increased expression of GFAP in the ventral tegmental area, a marker for astrocyte activation and reactivity [25]. Additionally, morphine dependence was associated with decreased expression of the astrocyte glutamate transporter GLT-1 in the striatum and thalamus [26]. Furthermore, pharmacological activation of glutamate transporters decreased drug-related behaviors associated with morphine, such as conditioned place preference [27]. Whether these effects are mediated indirectly, through neuronal signaling, or directly, through activation of astrocyte receptors, remains to investigated. The present demonstration of direct astrocyte responsiveness to opioids through activation of μ-opioid receptors suggests that these functional changes associated with morphine-related behaviors are mediated by astrocytes, which would suggest the participation of astrocytes in drug addiction behaviors.

One of the first studies investigating the physiological consequence of μ-opioid receptor activation on astrocytes found that activation of these receptors in the hippocampus led to astrocytic glutamate release through TREK-1-containing, two-pore potassium (K2P) channels [7]. In this case, μ-opioid receptor induced glutamate release was calcium independent [7]. Our present results, on the other hand, suggest that glutamate release in nucleus accumbens astrocytes occurs in a calcium-dependent manner. Further studies are required to elucidate whether there is heterogeneity across brain regions in regard to opioid-induced glutamate release from astrocytes [22,28] or there are multiple mechanisms of opioid-induced glutamate release from astrocytes in a particular brain region [29].

In the concept of the tripartite synapse [9], astrocyte calcium elevations are associated with gliotransmitter release, notably glutamate that can affect neuronal and synaptic activity. In accordance with many studies [9], it is widely accepted that SICs are generated by astrocytic glutamate release and results from activation of extrasynaptic NMDA receptors that contain the GluN2B subunit. Enhanced neuronal synchronization or neuronal excitability following SICs has been observed in the hippocampus and nucleus accumbens [20,30], indicating that astrocytes responding to opioids may influence local neuronal network activity in these brain regions. 

In summary, the current results demonstrate that opioids and opioid signaling impact astrocyte–neuron communication in the nucleus accumbens. Therefore, astrocytes may serve as a potential target for therapeutics designed to treat opioid use disorders. 

## Figures and Tables

**Table 1:** GFAP μ-opioid receptor expression quantification.

Condition	Cells with Co-Expression/Total # Cells	%
GFAP-mcherry + μ-opioid antibody	138/140	98.6

**Table 2:** AAV8–GFAP–mCherry–CRE viral vector specificity quantification.

Condition	Cells with Co-Expression/Total # Cells	%
AAV8–GFAP–mCherry–CRE + GFAP	270/287	94.1
AAV8–GFAP–mCherry–CRE + NeuN	11/136	8.1

**Table 3:** Summary statistics table.

Figure	*n* (# Animals)	Test	*t* Value	Z Statistic	*p* Value
1f	19 (8)	Two-tailed student’s paired *t*-test	−2.496	-	0.0225
2d	17 (7)	Two-tailed student’s paired *t*-test	−2.651	-	0.0174
3c	7 (3)	Two-tailed student’s paired *t*-test	0.294	-	0.779
3f	5 (3)	Two-tailed student’s paired *t*-test	0.535	-	0.621
5c	8 (2)	Two-tailed student’s paired *t*-test	−1.354	-	0.218
5f	9 (2)	Wilcox Signed Rank Test	-	0	0.789
6c	8 (2)	Wilcox Signed Rank Test	-	0	0.789
6f	6 (2)	Two-tailed student’s paired *t*-test	0.349	-	0.741
